# JS-K activates G2/M checkpoints through the DNA damage response and induces autophagy via CAMKKβ/AMPKα/mTOR pathway in bladder cancer cells

**DOI:** 10.7150/jca.86393

**Published:** 2024-01-01

**Authors:** Yuwan Zhao, Shanhong Lin, Wenfeng Zeng, Xinghua Lin, Xingzhang Qin, Bailiang Miu, Sheng Gao, Haokai Wu, Jianjun Liu, Xiaojun Chen

**Affiliations:** Laboratory of Urology, Affiliated Hospital of Guangdong Medical University, Zhanjiang, Guangdong 524001, China.

**Keywords:** Bladder cancer, NO prodrug, DNA damage, Cell arrest, Autophagy

## Abstract

The aim of this study was to investigate the effects of JS-K, a nitric oxide donor prodrug, on DNA damage and autophagy in bladder cancer (BCa) cells and to explore the potential related mechanisms. Through detecting proliferation viability, cell morphology observation and colony formation assay low concentrations of JS-K significantly inhibited BCa growth while having no effect on normal cells. JS-K induced an increase in the level of DNA damage protein γH2AX and a decrease in the level of DNA damage repair-related proteins PCNA and RAD51 in BCa cells, indicating that JS-K can induce DNA damage in BCa cells and inhibit DNA damage repair. JS-K induced G2/M phase block and calcium overload using flow cytometry analysis. Moreover, we also investigated the levels of cell G2/M cycle checkpoint-related protein and autophagy-associated protein by western blot. The results of our study demonstrated that JS-K induced BCa cells G2/M phase arrest due to upregulating proteins related to DNA damage-related G2/M checkpoint activation (p-ATM, p-ATR, p-Chk1, p-Chk2, and p-Cdc2) and down-regulation of Cyclin B1 protein. In addition, our study demonstrated that JS-K-induced autophagy in BCa cells was related to the CAMKKβ/AMPKα/mTOR pathway.

## Introduction

The incidence of bladder cancer (BCa), one of the most prevalent carcinomas, is second only to that of prostate cancer among all carcinomas of the urinary system 1, 2. Smoking may be a separate component in the development of BCa, which has increased dramatically in recent years due to population expansion and aging 3. Currently, surgery is the primary form of treatment for BCa, with bladder perfusion therapy and postoperative chemotherapeutic medications being supplemented 4, 5. However, its susceptibility to recurrence and drug resistance to chemotherapy have always been issues of concern to clinicians, so it is urgent to study new anti-BCa therapeutics.

DNA damage refers to endogenous or exogenous damage to DNA double-stranded molecules in cells, including chemical substances, ionizing radiation, reactive oxide species, and so on 6. DNA damage in tumor cells is a key mechanism of the killing effect of chemotherapy drugs, such as platinum-based chemotherapies 7. DNA double-strand break is a typical form of DNA damage 8. After DNA double-stranded molecules occur in cells under endogenous or exogenous stress, a series of downstream protein networks can be activated through the DNA damage response (DDR) pathway, including the activation of the DNA damage repair pathway and the activation of the cell cycle checkpoint, with the result leading to DNA damage, cell apoptosis and autoimmune clearance 9, 10. In addition, when cells are activated in response to DNA damage-induced cell cycle checkpoints, respectively, the cells may also be stagnant in the corresponding division cycle stage in order to obtain additional time for the repair of DNA.

The process of encasing deteriorating organelles and other hazardous chemicals in specialized membrane structures called autophagosomes and transporting them to lysosomes for breakdown is known as autophagy, which is an evolutionary highly conserved catabolic activity 12. Autophagy can be separated into macroautophagy, microautophagy, and molecular chaperone-mediated autophagy depending on the various package contents and delivery techniques 13. In a variety of diseases caused by the accumulation of misfolded proteins, a large number of macroautophagy disorders have been found, and the degradation of proteins by macroautophagy is the most widespread. Numerous research in recent years has demonstrated that autophagy and tumors are tightly connected and that autophagy regulates tumor development, proliferation, metastasis, and energy metabolism 14. Anti-tumor drugs based on the theoretical basis of regulating autophagy activity have been used in clinical treatment 15. However, the dual role of autophagy in cancer promotion and inhibition remains controversial. On the one hand, autophagy is considered to be a special protective mechanism of the tumor itself 16. Autophagy can promote the survival of tumors, the main mechanism of which is that tumor cells enhance their survival ability in the state of low oxygen and low nutrition by using the energy generated by autophagy 17. On the other hand, autophagy promotes the stability of the genome, lessens tissue damage and inflammation, and suppresses the accumulation of oncogenic p62 protein aggregates, thereby inhibiting the development, invasion, and metastasis of tumors and thus contributing to the suppression of cancer 18.

JS-K (C_13_H_16_N_6_O_8_, CAS-No., 205432-12-8) is a novel diazenediolate nitric oxide donor drug and its mechanism of action is that it can combine with Glutathione S-transferase (GSTs) widely distributed in tumors and release nitric oxide (NO) under the catalysis of GSTs 19. A high concentration of NO can cause DNA damage and accumulation of reactive oxygen species in tumor cells, and finally lead to tumor cell apoptosis 20. Current studies have shown that JS-K has an obvious inhibitory effect on various malignant tumors such as prostate cancer, liver cancer 22-23, and multiple myeloma 24, and is well tolerated by normal tissues and cells, making JS-K a promising anti-tumor drug. Therefore, JS-K is considered to be a promising anti-tumor drug.

We concentrated on the effectiveness and mechanism of JS-K on BCa cells in this study, which demonstrated that JS-K inhibited the proliferation of BCa cells (T24 and 5637) in a time-concentration dependent manner and had no discernible impact on normal cells (SV-HUC-1) at concentrations below 2 μM. We investigated the effect of JS-K on DNA damage in BCa cells, and found that JS-K could cause DNA damage in BCa cells and activate cell cycle checkpoints through the DDR pathway, leading to G2/M phase arrest. Furthermore, we discovered that JS-K led to calcium ion overflow in BCa cells and further activated the CAMKKβ/AMPKα/mTOR signaling pathway, which led to autophagy. Calcium ion overload may be the main mechanism of JS-K anticancer effect. Pretreatment of calcium ion chelating agent BAPTA can reduce the cytotoxicity and DNA damage of JS-K to BCa cells, and attenuated autophagy.

## Materials and Methods

### Cell culture

Human BCa cells (T24 and 5637) and Human ureteral epithelial cells (SV-HUC-1) were obtained from the Cell Bank of the Chinese Academy of Sciences of Shanghai. Both T24 and 5637 cells were cultured in the RPMI 1640 medium. SV-HUC-1 cells were cultured in Ham's F-12K (Kaighn's) medium. All mediums contained 10% fetal bovine serum (GIBCO, Thermo Fisher Scientific, Inc., Waltham, MA, USA) and 1% Penicillin-Streptomycin (Suolaibao Bio-Technology Co., Ltd., Shanghai, China) at 37°C and 5% CO_2_ humidity.

### Reagents and antibodies

The NO donor drug JS-K was obtained from Santa Cruz Biotechnology, Inc. (Dallas, TX, USA) and the Calcium ion chelating agent BAPTA was purchased from MedChemExpress (MCE, NJ, USA). JS-K and BAPTA were dissolved in dimethyl sulfoxide (DMSO) to concentrations of 5 mM and 20 mM respectively and stored in the refrigerator at -80 ℃. Antibodies against Phosphorylated histone H2AX (γH2AX), RAD51, PCNA, Phospho-ATM (p-ATM), Phospho-ATR (p-ATR), Phospho-Chk1 (p-Chk1), Phospho-Chk2 (p-Chk2), Phospho-Cdc2 (p-Cdc2), Cyclin B1, Phospho-CAMKKβ (p-CAMKKβ), Phospho-AMPKα (p-AMPKα), mTOR, Phospho-mTOR (p-mTOR), LC3B, Beclin 1, P62, GAPDH were purchased from Cell Signaling Technology, Inc. (Danvers, MA, USA). Antibodies against AMPKα were purchased from Cohesion Biotechnology Co., Ltd (Beijing, China). The Horseradish peroxidase conjugated IgG secondary antibodies were obtained from Shanghai Beyotime Biotechnology Co., Ltd.

### Cell proliferation assay

Cell proliferation was investigated by Cell Counting Kit-8 (CCK-8) (Apexbio, HOU, USA) assay. The T24, 5637, and SV-HUC-1 cells were seeded into a 96-well plate at 3×10^3^/well. When cell confluence reached about 60%, 100 μl of JS-K at different concentrations were added to the wells for 24 h, 48 h and 72 h and 0.1% DMSO was added as the negative control. After JS-K treatment for the corresponding time, 10 μl of CCK-8 regent was added to the medium of each well according to the instructions and the optical density (OD) values were detected after 2 h. The absorbance was measured at 450 nm using a Multiskan Ascent microplate photometer (EnSpire 2300 Multilabel Reader, PE, USA) at 450 nm.

### Colony formation assay

After T24, 5637 and SV-HUC-1 cells were treated with various concentrations of JS-K for 24 h, 800 live cells were collected and inoculated into 6-well plates. T24 and 5637 cells were incubated in an incubator at 37°C for 10 days while SV-HUC-1 cells were incubated for another 14 days. Then the cells were fixed with methanol for 20 min and stained with crystal violet (Beyotime, Shanghai, China). The macroscopic colonies were photographed and colony formation was subsequently calculated.

### Cell Cycle Assay

T24 and 5637 cells were treated with various concentrations of JS-K for 24 h. Then the cells were collected, washed with pre-cooled PBS, and then fixed in 70% pre-chilled ethanol overnight in a 4°C refrigerator. The next day, the cells were washed with pre-cooled PBS and stained with propidium iodide (10 µg/mL) according to the instruction manuals. The fluorescence intensity was measured by fluorescence-activated cell sorting.

### Intracellular calcium ion detection

Ca^2+^ indicator Fluo-4 (Fluo-4 AM) Assay Kit (Beyotime, Shanghai, China) was used to detect intracellular Ca^2+^ levels in T24 and 5637. Briefly, T24 and 5637 were inoculated at 3 × 10^5^ cells/well into 6-well plates and cultured overnight. When cell confluence reached approximately 60%-70%, the cells were treated with various concentrations of JS-K for 24 h. Cells were then collected and washed three times with pre-cooled PBS. Following the cells were resuspended in 0.5 ml of PBS solution containing 5 μM Fluo-4 AM at 37°C for 30 min. Finally, intracellular Ca^2+^ levels were analyzed using BD-FACSDiva 6.1 software.

### Immunofluorescence analysis of DNA damage

T24 and 5637 were inoculated at 1 × 10^5^ cells/dish into laser confocal dishes and cultured overnight, and then the cells were treated with various concentrations of JS-K for 24 h. The specific experimental steps were referred to previous article 25 and the cells were finally photographed with a laser scanning confocal microscope at 100× magnification.

### Immunofluorescence analysis of autophagy

CYTO-ID Autophagy Detection Kit (Enzo Life Science, NY, USA) was used to detect the occurrence of autophagy. T24 and 5637 were seeded into the laser confocal dishes at 1×10^5^ cells/dish and cultured overnight, and then the cells were treated with various concentrations of JS-K for 24 h and Rapamycin (500 nM) as a positive control. According to the instruction manuals, the cells were washed twice with 1× assay buffer containing 5% FBS before being stained with CYTO-ID green detection reagent for 30 min at 37°C. Subsequently, the cells were washed with 1× assay buffer containing 5% FBS and fixed with 4% paraformaldehyde for 20 min. Finally, the cells were photographed with a laser scanning confocal microscope at 100× magnification.

### Western blot analysis

T24 and 5637 cells were treated with different concentrations of JS-K for 24 h and then washed three times with pre-cooled PBS. Whole proteins were extracted by adding radioimmunoprecipitation assay (RIPA) buffer (Beyotime, Shanghai, China) supplemented with 1 mM phenylmethanesulfonyl fluoride (PMSF) (Beyotime, Shanghai, China). About 20 μg of the cell lysate was separated by 12% SDS-PAGE and blotted onto a PVDF membrane (EMD Millipore, Billerica, MA, USA). After blocking with 5% non-fat milk, the membranes and primary antibodies were incubated overnight at 4 ℃. The antibody diluent buffer (Beyotime, Shanghai, China) was used to dilute primary antibody γH2AX (#2577S; dilution 1: 1,000), RAD51 (#8875S; dilution 1: 1,000), PCNA (#2586S; dilution 1: 1,000), p-ATM (#5883; dilution 1: 1,000), p-ATR (#2853;dilution 1: 1,000), p-Chk1 (#2348P; dilution 1: 1,000), p-Chk2 (#2661P; dilution 1: 1,000), p-Cdc2 (#9111P; dilution 1: 1,000), Cyclin B1 (#12231T; dilution 1: 1,000), p-CAMKKβ (#12716T; dilution 1: 1,000), p-AMPKα (#2535S; dilution 1: 1,000), mTOR (#2983S; dilution 1: 1,000), p-mTOR (#2971S; dilution 1: 1,000), LC3B (#2775S; dilution 1: 1,000), Beclin 1 (#3738; dilution 1: 1,000), P62 (#8025S; dilution 1: 1,000), GAPDH (#2118S; dilution 1: 1,000). Subsequently, the membrane was incubated with IgG-HRP secondary antibody (Beyotime, Shanghai, China) and an enhanced chemiluminescence kit (EMD Millipore, Billerica, MA, USA) with Tanon 5200 chemiluminescent imaging system (Shanghai, China).

### Transmission electron microscopy

T24 and 5637 cells were treated with 2 μM JS-K for 24 h and harvested and placed in 0.1m sodium phosphate buffer and fixed with 2.5% glutaraldehyde at 37℃ for 2 h, and then dehydrated with graded ethanol series and embedded. Ultra-thin sections of about 70 nm were mounted on nickel grids. The autophagosomes were visualized using the 120-kV Jeol electron microscope (JEM-1400; JEM, Peabody, MA, USA) and the images were collected by the Gatan-832 digital camera (GATAN, Pleasanton, CA, USA).

### Subcutaneous xenograft nude mouse model

6-week-old male BALB/c nude mice were obtained from the specific pathogen-free animal laboratory of Southern Medical University. Well-grown 5637 cells (5 x 10^6^ cells) were resuspended and injected subcutaneously into thymopathy mice. Following that, we took care of nude mice every day and carefully observed and recorded the size of the tumor volume and measured the tumor growth weekly. When the tumor volume reached 50 mm^3^, treatment with JS-K was initiated by tail vein injection of a dose (10 mg/kg mice (n=5)) and tumor volume was measured once a week. At the end of the experiment, the animals were sacrificed and the xenograft tumors were extracted to complete follow-up experiments.

### Histology and immunohistochemistry

The xenograft tumors were fixed in formalin overnight and following paraffin embedding. Paraffin blocks were sectioned and stained with hematoxylin and eosin (H & E). The immunohistochemistry (IHC) assay was performed using the antibodies LC3A/B (Cell Signaling Technology), γH2AX (Abcam), Rad51 (Abcam), P62 (Abcam), Ki67 (Abcam), P21 (Abcam) and Cyclin B1 (Abcam). All IHCs were performed as described previously. Images were measured by Image-pro-plus software (Version 6.0) and fluorescence intensity was calculated based on average staining intensity and positively stained cells percentage. Both the percentage of positive cells and the intensity of protein expression were combined to calculate a total score of protein expression.

### Statistical analysis

Data analysis in this study was performed using SPSS 19.0 software for statistical analysis, and the measurements data were expressed as mean ± standard deviation. One-way analysis of variance was used for comparison between multi-group differences and the least significant difference method (LSD method) was used for statistical analysis for pairwise comparisons. The comparison between the two groups was performed on the basis of the Student's t-test, *P < 0.05, **P < 0.01, ***P < 0.001 were considered statistically significant.

## Result

### The effects of JS-K on the proliferation of BCa Cells

**Figure 1:**
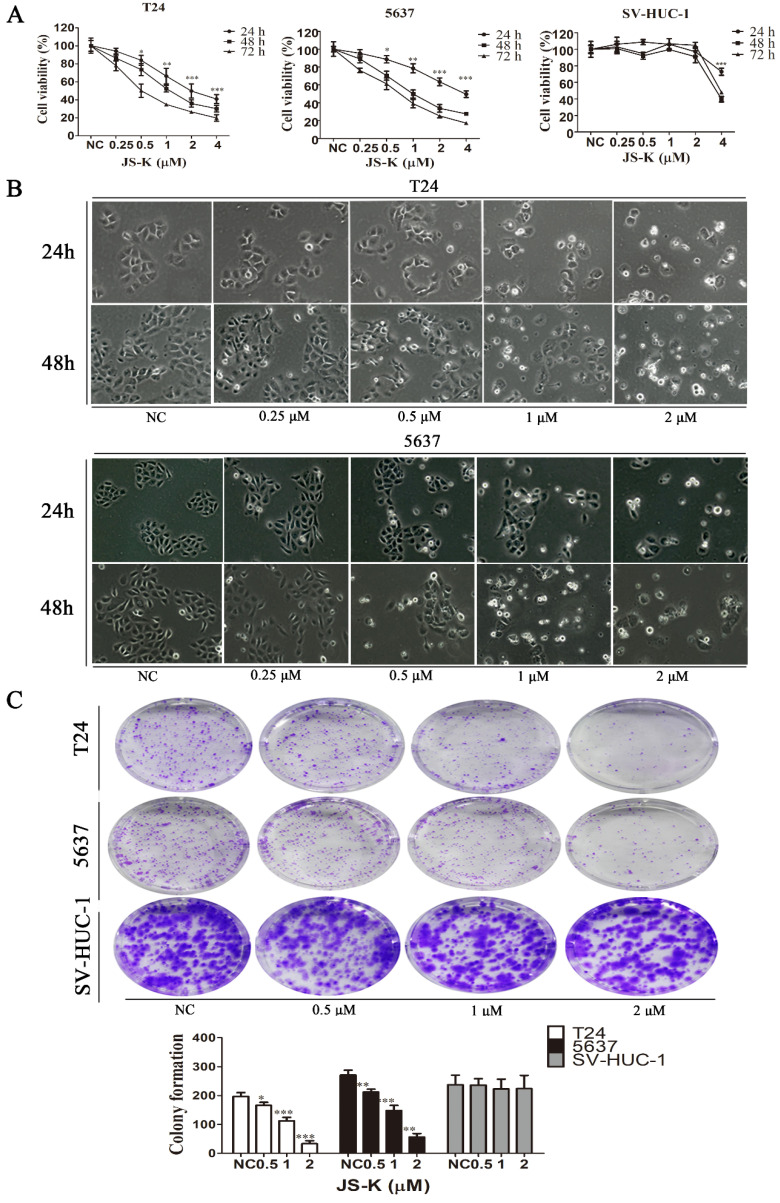
** Effects of JS-K on the proliferation of BCa cells.** The CCK-8 assay was performed to determine BCa cell viability treated with 0.25, 0.5, 1, 2, and 4 μM JS-K. (A) JS-K inhibited the cell viability of BCa cell lines T24 and 5637 in a concentration-and time-dependent manner, and low concentrations of JS-K did not inhibit bladder normal cell line SV-HUC-1. (B) BCa cell lines T24 and 5637 were treated with different concentrations of JS-K for 24 and 48 h after treatment by phase contrast microscopy (magnification, x10) to induce morphological changes. (C) The ability of JS-K to inhibit colony formation in BCa cell lines T24 and 5637. All of the data are presented as the mean ± SD based on one-way ANOVA (n=3, *P < 0.05, **P < 0.01, ***P < 0.001 versus negative control).

The effect of JS-K on the proliferation of BCa cells was investigated using the CCK-8 assay, where T24 and 5637 cells were treated at JS-K concentrations (0, 0.25, 0.5, 1, 2, and 4 μM) for 24 h, 48 h, 72 h. The low concentrations of JS-K (0.5 μM) significantly inhibited the proliferation of BCa cells in a time- and concentration-dependent manner. However, for normal cell line SV-HUC-1, low concentrations of the drug did not induce significant proliferation inhibition, but only at concentrations greater than or equal to 4 μM (Figure 1A). Furthermore, morphological changes in BCa cells were observed using phase contrast microscopy, and in agreement with the results of CCK-8, there were no morphological changes in BCa cells with 0.25 μM JS-K. When the concentration of JS-K was greater than or equal to 0.5 μM, morphological changes began to occur in BCa cells, with a decrease in the number of cells and a decrease in intercellular contacts (Figure 1B). We further investigated the effect of JS-K on the cloning ability of BCa cells. JS-K inhibited the cloning ability of BCa cells in a concentration-dependent manner, with no significant effect on normal cells (Figure 1C).

### JS-K induces BCa cell's DNA damage and inhibits DNA damage repair

**Figure 2:**
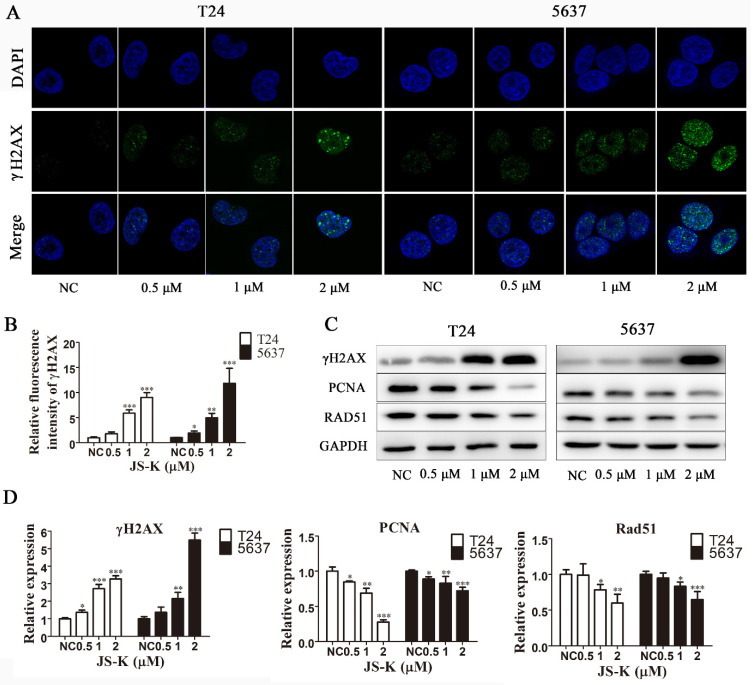
** JS-K induced DNA breakage and inhibited DNA damage repair on BCa cell lines T24 and 5637.** (A and B) JS-K increased the foci of DNA damage-associated protein γH2AX on BCa cell lines T24 and 5637 in a concentration-dependent manner, as determined by immunofluorescence analysis. The images were captured by laser scanning confocal microscope at 100× magnification and were determined by immunofluorescence analysis. (C and D) Western blot assay was used to determine the effects of JS-K on DNA damage marker protein γH2AX and DNA damage repair-associated protein PCNA and Rad51 on BCa cell lines T24 and 5637. All of the data are presented as the mean ± SD based on one-way ANOVA (n=3, *P < 0.05, **P < 0.01, ***P < 0.001 versus negative control).

To explore the mechanism of action of JS-K, we used immunofluorescence analysis and western blot assay to investigate whether JS-K activated the DNA damage response in BCa cells. The levels of the DNA damage marker protein γH2AX and its foci levels increased significantly with increasing JS-K concentrations, suggesting that JS-K induces DNA double-strand breaks in BCa cells and further leads to DNA damage (Figure 2A and 2B). In addition, we investigated the effect of JS-K on DNA damage repair-associated protein PCNA and RAD51. The results showed that JS-K inhibited the levels of PCNA and RAD51 with increasing concentrations, suggesting that JS-K may be associated with the inhibition of DNA damage repair responses in BCa cells (Figure 2C and 2D).

### JS-K activates DNA damage cheakpoint and leads to G2/M phase arrest

**Figure 3:**
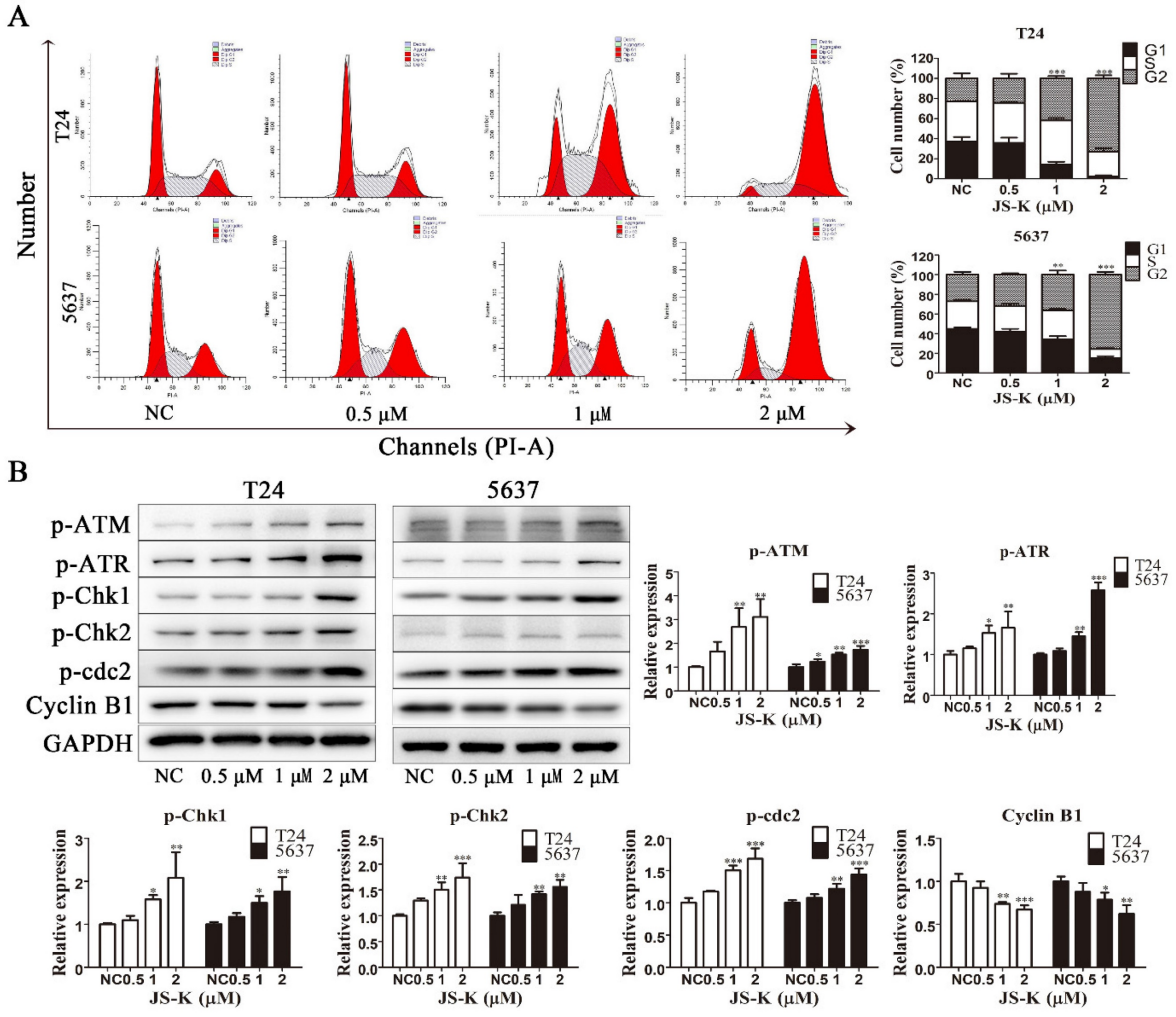
** JS-K induced G2/M cell cycle arrest and activated DNA damage cheakpoint on BCa cell lines T24 and 5637.** (A) Cell cycle analysis was performed to determine the effects of JS-K on BCa cells by fluorescence-activated cell sorting. (B) The DNA damage cheakpoint and G2/M cell cycle arrest relative protein p-ATM, p-ATR, p-Chk1, p-Chk2, p-cdc2 and Cyclin B1 was detected by western blot assay. All of the data are presented as the mean ± SD based on one-way ANOVA ( n=3, *P < 0.05, **P < 0.01, ***P < 0.001 versus negative control).

To investigate the effect of JS-K on the cell cycle in BCa cells, we used flow cytometry to analyze the cell cycle by PI-labeled DNA content. As the Fig. 3A shows, treatment with increasing concentrations of JS-K resulted in the accumulation of the BCa cell cycle in the G2/M phase, indicating G2/M phase arrest. Subsequently, to further investigate the possible mechanism leading to BCa cells G2/M arrest, we found the mechanism may be relative to the activation of DNA damage cheakpoint. After treatment with increasing concentrations of JS-K, the protein expression levels of p-ATM, p-ATR, p-Chk1, p-Chk2, and p-Cdc2 increased and Cyclin B1 was decreased (Figure 3B). These results indicated that JS-K induced DNA damage in BCa cells and leads to activation of G2/M checkpoint, which led to cell cycle G2/M arrest.

### JS-K causes intracellular calcium ion overload in BCa cells

**Figure 4:**
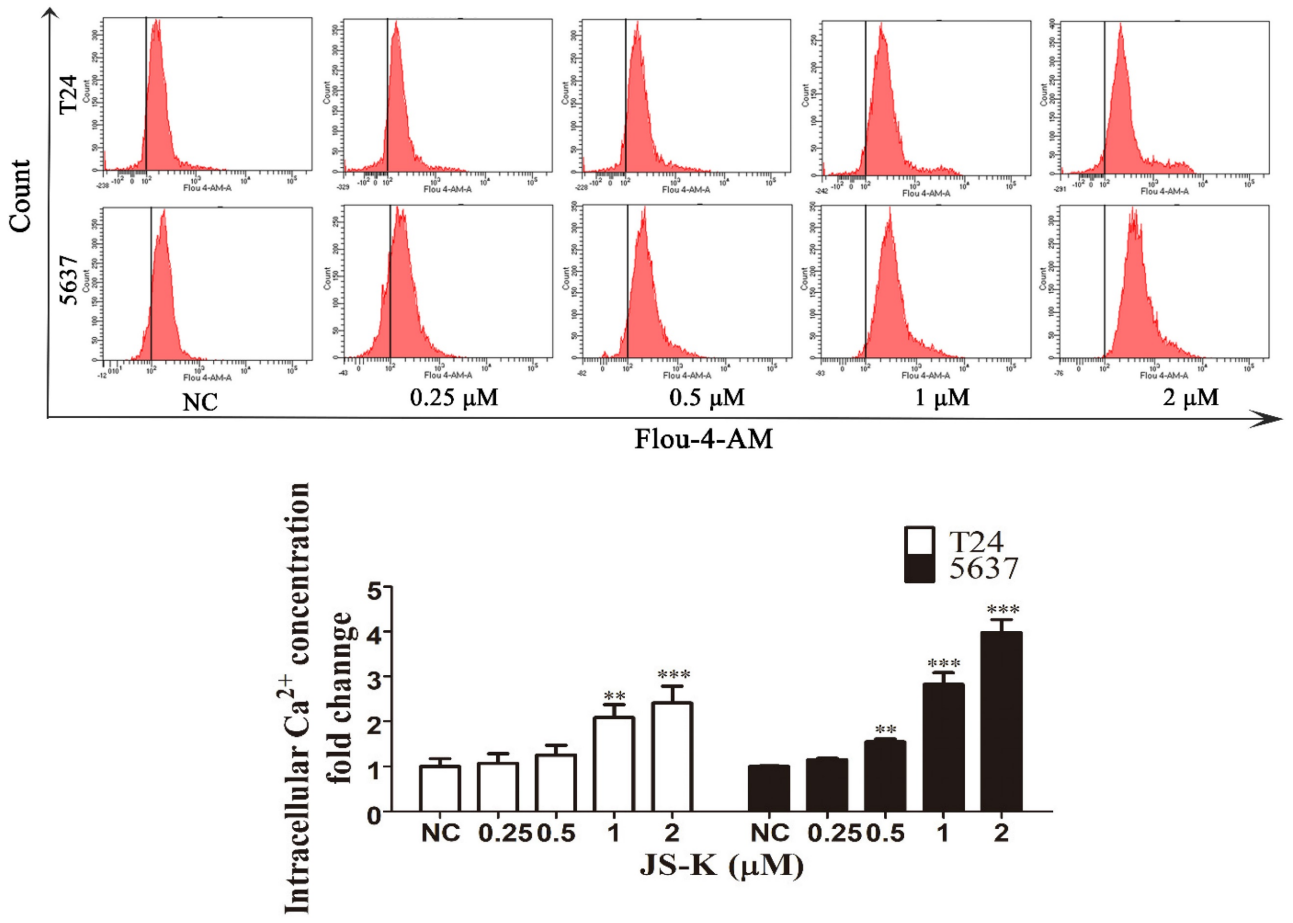
** JS-K induced intracellular calcium ion overload in BCa cell lines T24 and 5637.** The level of intracellular calcium ion was determined using the Fluo-4 AM calcium ion fluorescent probe and analyzed by the flow cytometer. The data are presented as the mean ± SD based on one-way ANOVA ( n=3, *P < 0.05, **P < 0.01, ***P < 0.001 versus negative control).

The Fluo-4 AM calcium ion fluorescent probe was used to detect the calcium ion level of JS-K in BCa cells by flow cytometry. After T24 and 5637 cells were treated with different concentrations of JS-K (0, 0.25, 0.5, 1, and 2 μM) treated with T24 and 5637 cells for 24 h, we found that the fluorescent level of calcium ions in the cells increased with the increase of JS-K concentration, which indicated that JS-K could cause calcium ion overload in BCa cells (Figure 4).

### JS-K induces autophagy via CAMKKβ/AMPKα/mTOR pathway in BCa cells

**Figure 5:**
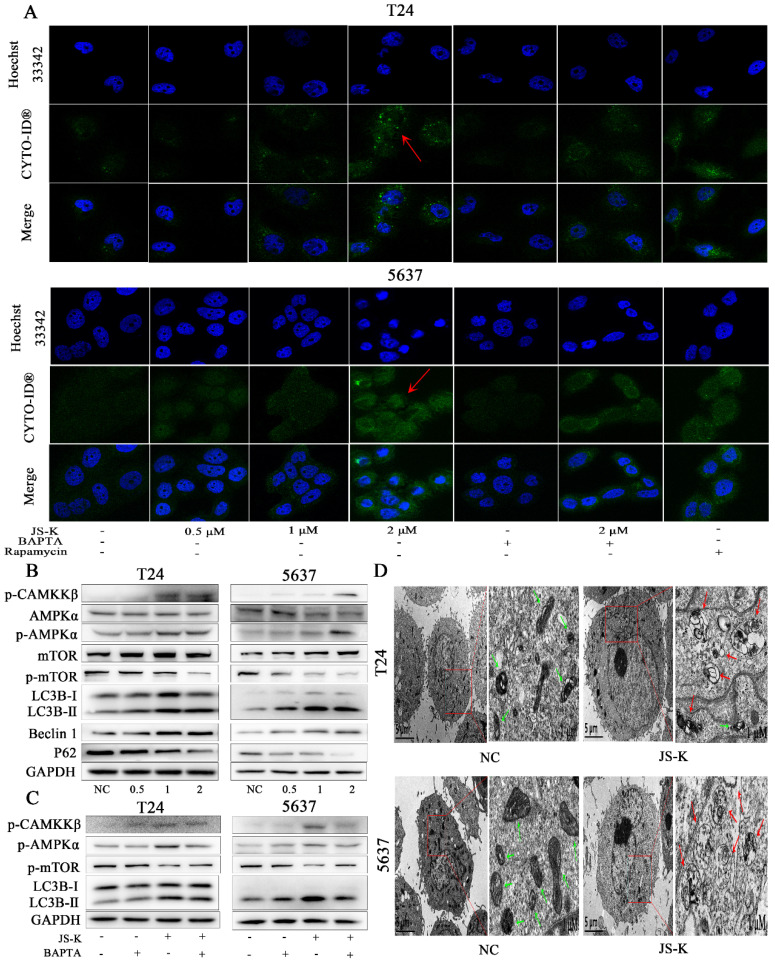
** JS-K induced autophagy via CAMKKβ/AMPKα/mTOR pathway in BCa cells.** (A) Laser scanning confocal microscopy observed that JS-K increased the fluorescent signal of autophagy. (B and C) Expression of CAMKKβ/AMPKα/mTOR pathway and autophagy-related proteins (p-CAMKKβ, p-AMPKα, p-mTOR, LC3B Ⅱ/Ⅰ, Beclin 1 and P62) were determined by western blot assay. (D) Increased autophagosomes formation and mitochondrial swelling, deformation, and distortion were observed under transmission electron microscopy (the red arrows represent autophagosomes and the green arrows represent mitochondria).

To more thoroughly examine the potential link between intracellular calcium ion overload and autophagy in BCa cells, the production of autophagy was directly observed utilizing the CYTO-ID Autophagy Detection Kit by immunofluorescence analysis to determine the foci level. The stronger fluorescent signal could be detected after JS-K processing (Figure 5A). We discovered that p-protein CAMKKβ's expression level increased, and as a result, its downstream protein AMPK α was also activated and mTOR activity was blocked. This led us to further examine the underlying process. The results of a western blot assay used to identify autophagy-related proteins showed that P62 expression levels fell while LC3B II and Beclin 1 expression levels increased (Figure 5B). In addition, we found that pre-treated with calcium ion chelator BAPTA 10 μM for 1 h could reduce the level of intracellular calcium ion overload caused by JS-K and decrease the protein expression level of p-CAMKKβ, p-AMPKα and LC3B Ⅱ while the inhibition of mTOR was decreased (Figure 5C). In addition transmission electron microscopy was used to examine the morphology of BCa cells that had been exposed to js-k for 24 hours. We found that JS-K induced a massive accumulation of autophagosomes in T24 and 5637 cells, suggesting that the final step of autophagy was disrupted, autophagosomes fuse with lysosomes, and cellular contents were degraded (Figure 5D). These results indicated that JS-K induced autophagy may be related to CAMKKβ/AMPKα/mTOR pathway in BCa cells.

### Pretreatment with calcium chelator BAPTA attenuates the cytotoxicity and DNA damage of JS-K in BCa cells

**Figure 6:**
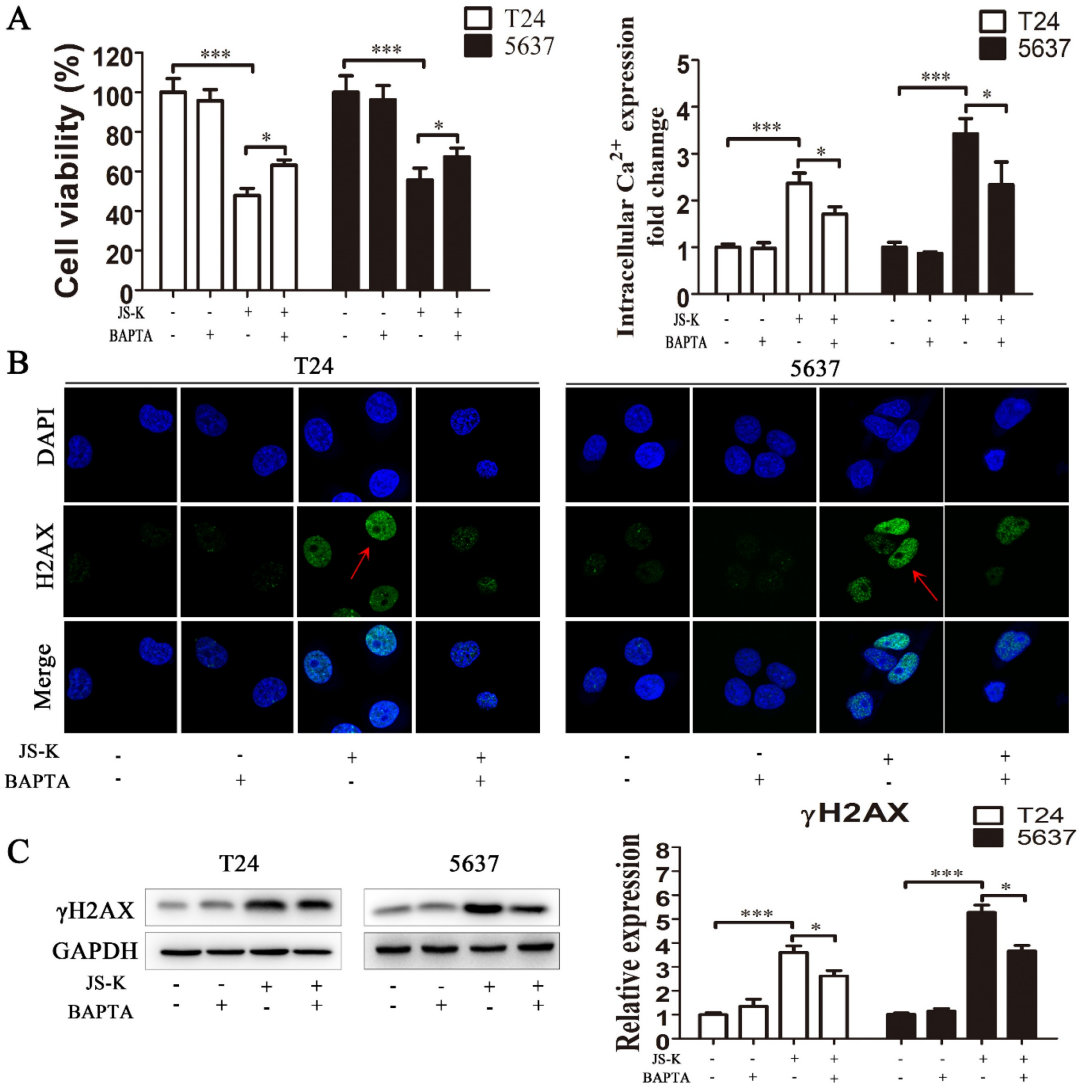
** Reduced intracellular calcium levels counteracted the cytotoxic effects of JS-K in BCa cells.** (A) The CCK8 assay was performed to determine the cell viability of pretreatment with calcium chelator BAPTA (n=3, *P < 0.05, ***P < 0.001). (B) The Fluo-4 AM calcium ion fluorescent probe assay was performed to determine the level of intracellular calcium ion of pretreatment with calcium chelator BAPTA (n=3, *P < 0.05, ***P < 0.001). (C) The immunofluorescence analysis was performed to determine the foci of DNA damage-related protein γH2AX of pretreatment with calcium chelator BAPTA. (D) The western blot assay was performed to determine the expression level of DNA damage marker protein γH2AX of pretreatment with calcium chelator BAPTA (n=3, *P < 0.05, ***P < 0.001). The column are presented as the mean ± SD based on the Students t-test.

After pretreatment with 10 μM BAPTA for 1 h, we performed the CCK-8 test to identify the suppression of cell growth brought on by JS-K, suggesting that lowering the level of intracellular calcium ion reduced the cytotoxic effect of JS-K on BCa cells (Figure 6A and 6B). We performed immunofluorescence analysis and western blot to assess DNA damage alterations in response to the calcium ion overload generated by JS-K on BCa cells. Pretreatment of BCa cells with 10 μM BAPTA for 1 h significantly reduced the level of DNA damage marker protein γH2AX and its foci level caused by JS-K. These results suggested that the effects of JS-K on DNA damage and proliferation inhibition in BCa cells may be related to intracellular calcium overload to a certain extent (Fig. 6C and 6D).

### JS-K exhibited a strong therapeutic effect on the xenograft mouse model

**Figure 7:**
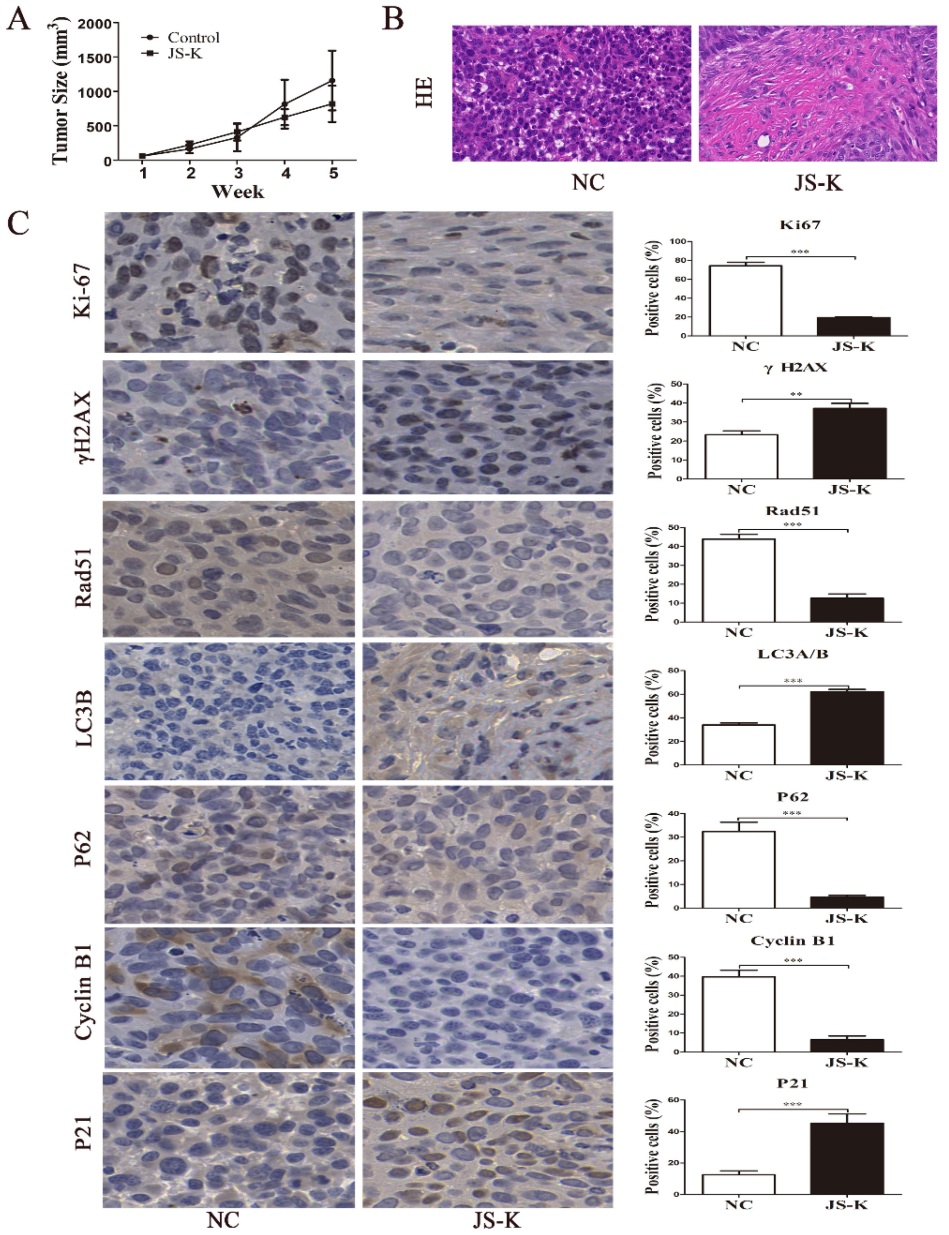
** Effects of JS-K on the tumors in a xenograft mouse model.** (A) Tumor volume calculation. (B) H&E staining of all xenografts. (C) The IHC assays of xenograft tumors were performed to determine the expression of LC3B, γH2AX, P21, Rad51, Ki67, P62, and Cyclin B1 (scale bar = 10 μm). The columns are presented as the mean ± SD based on the Students t-test (n=3, **P < 0.01, ***P < 0.001 versus negative control).

To investigate the therapeutic effect of the JS-K treatment *in vivo*, we constructed a xenograft mouse model of 5637 cell. According to the findings, increasing the therapeutic dose of JS-K caused a change in the tumor tissue's structure and a decrease in the size of the xenograft tumor in nude mice (Figure 7A and 7B). IHC assay was carried out to further confirm that JS-K caused autophagy and cell cycle arrest *in vivo*. As the results show, JS-K increased the expression of LC3B, γH2AX, and P21, but decreased the expression of Rad51, Ki67, P62, and Cyclin B1 (Figure 7C). These indicated that JS-K-induced cell cycle arrest and autophagy in BCa cells were at the animal level, which was consistent with the cellular level.

## Discussion

Traditional chemotherapy and radiotherapy used in tumor treatment usually cause DNA damage to tumor cells through chemical or physical radiation and then lead to chromosome instability 26. Core histone γH2AX is more phosphorylated when DNA double-strand breaks occur in cells, which allows γH2AX to be utilized as a marker protein for DNA damage and to reflect DNA damage in an indirect manner 27. In addition, proliferating nuclear antigen (PCNA) is the central molecule at the intersection of DNA replication and DNA repair and plays an important role in the process of DNA replication and repair in cells 28. In the DNA damage repair response pathway that is initiated upon DNA damage, Rad51 is a key player in the process of homologous recombination repair 29-30. Therefore, PCNA and RAD51 play an important role in DNA damage repair. In our study, we examined the effects of various concentrations of JS-K on DNA damage in BCa cells. Under the observation of the fluorescence microscope, the concentration of JS-K steadily enhanced the fluorescence density and intensity of the DNA damage marker protein γH2AX. The same results were also seen in Western blot analysis. With the increase of JS-K treatment concentration, the gray value of DNA damage marker protein γH2AX was enhanced, while the expression of DNA damage repair proteins PCNA and Rad51 was inhibited, suggesting that JS-K could cause DNA damage in BCa cells and inhibit DNA damage repair.

Activation of cell cycle checkpoints is a key component of the DNA damage repair response pathway 31. Cdc2 and Cyclin are dynamic regulators of cell cycle progression. Cyclin B1 is associated with the G2/M phase checkpoint and is capable of binding to Cdc2 to form a complex, which plays an important role in the progression from G2 phase to M phase 32. The complex formed by CyclinB1 and Cdc2 is the engine molecule for cells to transfer from G2 phase to M phase, and the activation of Cdc2 is the key to driving the process from G2 phase to M phase 33. Cell cycle arrest can result from phosphorylating Cdc2 at Try15, which can inhibit Cdc2 activity 34. The findings of our investigation demonstrated that when JS-K was larger than μ1 M, BCa cells' cell cycle was halted in the G2/M phase. The western blot showed that the expression level of p-Cdc2 was increased while the expression level of Cyclin B1 was decreased, indicating that JS-K caused the phosphorylation of Cdc2 protein in BCa cells, thereby reducing the activity of Cdc2 and the expression level of Cyclin B1and further causing the cell cycle arrest. In addition, ATR/ Chk1 and ATM/ Chk2 protein kinase pathways are two major signaling pathways activated by DNA damage repair pathways, and activation of ATR and ATM is able to activate phosphorylation of Chk1 and Chk2, respectively 35. Checkpoint kinases 1 and 2 (Chk1 and Chk2) are key mediators of the G2/M phase cell cycle checkpoint, and the activation of Chk1 and Chk2 is capable of promoting cell cycle arrest 36. In order to further investigate the related mechanism of JS-K causing cycle arrest of BCa cells, we found that the expression levels of p-ATM, p-ATR, p-Chk1 and p-Chk2 increased, suggesting that the G2/M phase arrest induced by JS-K may be related to the DNA damage repair response pathway.

The dual role of autophagy in either promoting or inhibiting tumor growth has been a major study subject in recent years 37. Previous studies had shown that JS-K caused apoptosis of breast cancer 38 and ovarian cancer 39 and also promoted the process of autophagy. Therefore, autophagy may be one of the mechanisms of JS-K's inhibition of tumors, and the increased level of autophagy may promote the death of tumor cells. We thought about whether the inhibitory impact of JS-K on BCa cells was connected to the autophagic process based on the aforementioned points of view. Previous research conducted in our lab had demonstrated that JS-K caused BCa cells to accumulate reactive oxygen species (ROS) before inducing apoptosis 40. With the increase of intracellular ROS level, the endoplasmic reticulum that stores calcium ions in the cell could release calcium ions as well as the influx of calcium ions in the internal environment, thus causing calcium ion overload 41. CAMKKβ is an important member of the calcium/calmodulin-activated protein kinase family and is activated when intracellular Ca2+ levels are increased 42. In addition to controlling cell proliferation, protein synthesis, and the cell cycle, AMPK is a crucial regulator of cellular energy homeostasis 43. Activation of CAMKKβ activates downstream AMPK, which in turn inhibits mTOR activity 44. It is well known that mTOR is a negative regulator of autophagy, and when the activity of mTOR is inhibited, it is able to induce the activation of autophagy 45. In our study, we found that JS-K caused calcium ion overload and induced the process of autophagy. In addition, the protein level of p-CAMKKβ, p-AMPKα, LC3BⅡ/Ⅰ increased and p-mTOR decreased, which indicated that JS-K inducing the process of autophagy was related to CAMKKβ/AMPKα/mTOR pathway. Additionally, the level of the calcium ion overload brought on by JS-K and the subsequent reduction in the cytotoxic effect of JS-K were both reduced by pretreatment with the calcium ion chelating agent BAPTA. The DNA damage marker protein γH2AX and the protein associated with the CAMKKβ/AMPKα/mTOR pathway were suppressed by the drop in intracellular calcium ion levels, further weakening the effects of JS-K on DNA damage and the autophagy process in BCa cells. These revealed that the effects of JS-K on proliferation inhibition, DNA damage and the process of autophagy in BCa cells may be related to intracellular calcium overload to a certain extent.
